# Increased expression of cathepsin D is required for L1-mediated colon cancer progression

**DOI:** 10.18632/oncotarget.27155

**Published:** 2019-08-27

**Authors:** Sayon Basu, Sanith Cheriyamundath, Nancy Gavert, Thomas Brabletz, Gal Haase, Avri Ben-Ze’ev

**Affiliations:** ^1^Department of Molecular Cell Biology, Weizmann Institute of Science, Rehovot 7610001, Israel; ^2^Experimental Medicine I, Nikolaus-Fiebiger-Center for Molecular Medicine, University of Erlangen-Nuernberg, Erlangen 91054, Germany

**Keywords:** L1, cathepsin D, colon cancer, metastasis, Wnt/*β*-catenin signaling

## Abstract

Hyperactivation of Wnt/β-catenin target genes is considered a key step in human colorectal cancer (CRC) development. We previously identified the immunoglobulin-like cell adhesion receptor L1 as a target gene of β-catenin/TCF transactivation that is localized at the invasive edge of CRC tissue. Using gene arrays, we discovered a number of downstream target genes and signaling pathways conferred by L1 overexpression during colon cancer progression. Here, we have used a proteomic approach to identify proteins in the secretome of L1-overexpressing CRC cells and studied the role of the increase in the aspartate protease cathepsin D (CTSD) in L1-mediated colon cancer development. We found that in addition to the increase in CTSD in the secretome, the RNA and protein levels of CTSD were also induced by L1 in CRC cells. CTSD overexpression resulted in elevated proliferation under stress and increased motility, tumorigenesis and liver metastasis, although to a lesser extent than after L1-transfection. The suppression of endogenous CTSD in L1-expressing cells blocked the increase in the proliferative, motile, tumorigenic and metastatic ability of CRC cells. Enhancing Wnt/β-catenin signaling by the inhibition of GSK3β resulted in increased endogenous CTSD levels, suggesting the involvement of the Wnt/β-catenin pathway in CTSD expression. In human CRC tissue, CTSD was detected in epithelial cells and in the stromal compartment at the more invasive areas of the tumor, but not in the normal mucosa, indicating that CTSD plays an essential role in CRC progression.

## INTRODUCTION

The aberrant activation of Wnt/β-catenin signaling in colorectal cancer (CRC) cells is considered an early and critical step in tumorigenesis [1, 2]. We identified members of the L1CAM (L1) family of immunoglobulin-like cell adhesion receptors (L1 and NrCAM) as Wnt/β-catenin target genes that are activated during CRC development [3, 4] and detected L1 at the invasive front of CRC tissue [3]. We showed that the overexpression of L1 in CRC cells confers enhanced motility, tumorigenesis and metastasis to the liver, the most frequent site of CRC metastasis [3, 5]. L1 is well-known for its important roles in nerve cells, playing a key role during the dynamic processes of brain development, including neuronal and axonal growth, axonal pathfinding and fasciculation [6, 7]. Inactivating point mutations in L1 result in severe developmental neurological diseases such as X-linked hydrocephalus, MASA syndrome and L1 syndrome [7, 8] and block the tumorigenic-metastasis capacity conferred by L1 [9]. Using gene-arrays, we identified downstream target genes and pathways of L1-mediated signaling that lead to enhanced CRC progression [9–14]. Among them we found secreted proteins and colonic stem cell signature genes [12–14]. To identify proteins that are involved in conferring increased CRC development by L1, we conducted a proteomic analysis of the secretome in L1-expressing CRC cells. In this study, we identified the lysosomal aspartic protease cathepsin D (CTSD) among the proteins whose levels are increased in the secretome of L1-expressing CRC cells and studied the role of CTSD in L1-mediated CRC tumorigenesis.

## RESULTS

### Induction of cathepsin D expression in human colon cancer cells after transfection with L1

**Figure 1:**
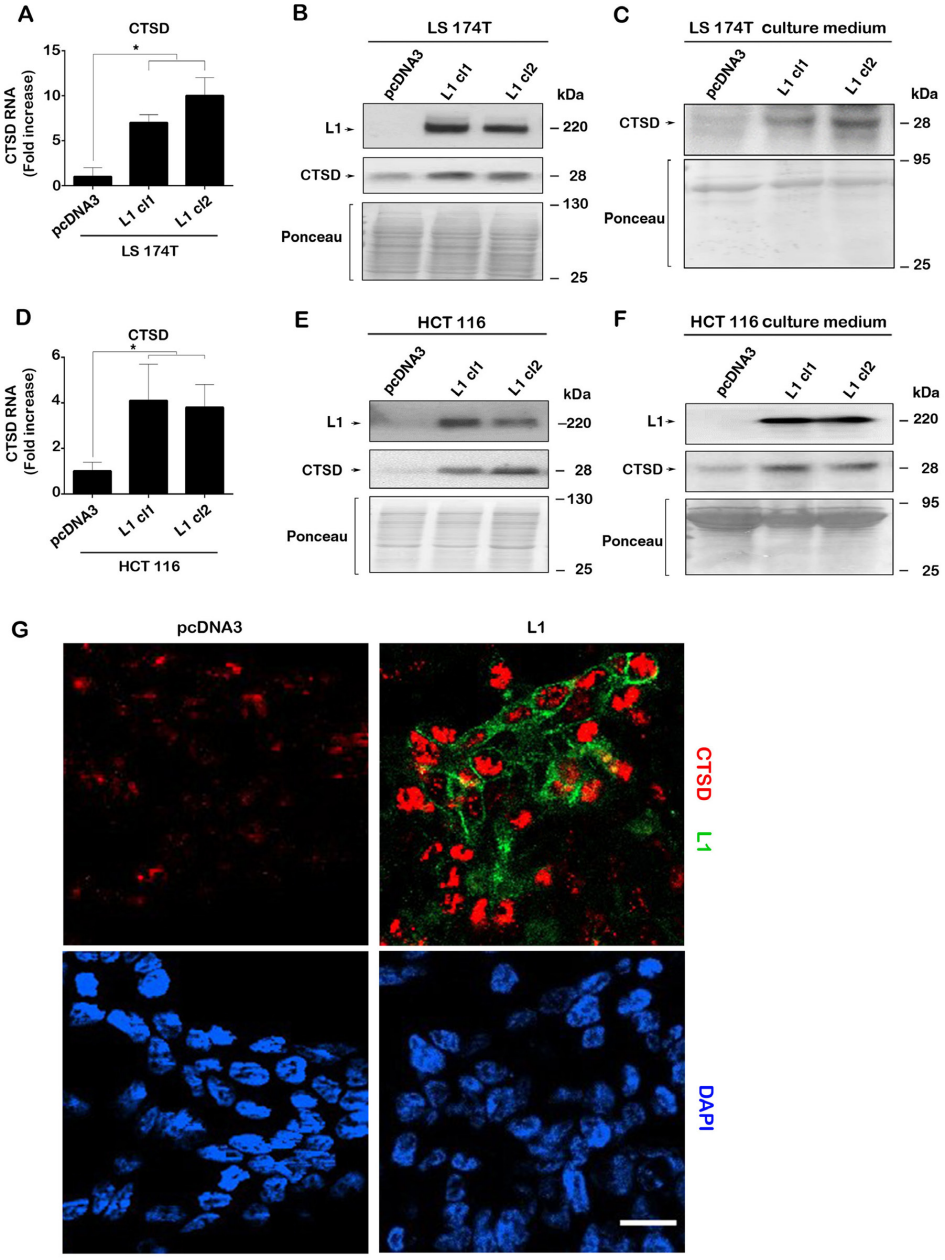
L1 overexpression in human CRC cell lines induces the expression of CTSD. (**A**) The levels of CTSD RNA (determined by RT-PCR) in L1-expressing LS 174T CRC cell clones are higher than in control pcDNA3-transfected cells. (**B**) Western blotting showing that CTSD protein levels are higher in L1-expressing CRC cells compared to control (pcDNA3 transfected cells) and (**C**), secreted CTSD levels in the culture medium are higher in L1-expressing LS 174T CRC cells. The same findings were seen in the human HCT 116 CRC cell line transfected with L1 that showed increased, (**D**) CTSD RNA, (**E**) CTSD protein and (**F**) CTSD protein secreted into the culture medium. (**G**) Endogenous CTSD immuno-staining (red) was much stronger in L1-transfected LS 174T cells (green), compared to control LS 174T cells (pcDNA3). Nuclei were stained with DAPI (blue). The bar represents 20 **μ**m. Ponceau staining of the western blots served for determining equal loading of the gels.

We wished to identify proteins that mediate the increase in the tumorigenic and metastatic capacity of colon cancer cells after their transfection with L1 [3, 5]. Proteins secreted into the culture medium of LS 174T CRC cells before and after stable transfection with L1 were analyzed by mass spectrometry and proteins displaying a more than 10-fold increase in their amount after L1 expression in these cells are shown in Table 1. In this study, we have chosen to focus on the aspartic protease cathepsin D (CTSD) because of its reported association with cancer development [15–18]. We found that in addition to the increase in CTSD among the proteins secreted into the cell culture medium (Table 1 and Figure 1C), CTSD RNA expression (Figure 1A) and protein levels (Figure 1B) were also elevated following the stable expression of L1 in individual LS 174T cell clones. A parallel increase in the pro-CTSD precursor was also observed in both the cell layer and in the culture medium of LS 174T cells (Supplementary Figure 1A and 1B).

**Table 1:** Proteins secreted at higher level from LS 174T cells expressing L1 compared to control LS 174T cells

Gene Symbol	Description	Fold Change
CALCA	Calcitonin-related polypeptide alpha	400
HADHB	Hydroxyacyl-CoA dehydrogenase trifunctional multienzyme complex beta	382.8
MUC2	Mucin 2	82.6
BGN	Biglycan	68
SMOC2	SPARC related modular calcium binding protein 2	53
CTSD	Cathepsin D	30.3
VCAN	Versican	18.1
SEMA3B	Semaphorin 3B	15.5
ADPRHL2	ADP-ribosylhydrolase like 2	11

Mass spectrometric analysis of proteins secreted into the cell culture medium from LS 174T cells stably expressing L1 compared to LS 174T cells transfected with pcDNA3. Proteins whose level was increased >10 fold in L1-expressing cells are shown.

In addition to the LS 174T CRC cell line, the stable expression of L1 in the HCT 116 human CRC cell line also resulted in increased expression of CTSD RNA (Figure 1D) and protein (Figure 1E) and an increase among the proteins secreted into the culture medium (Figure 1F). This induction in CTSD expression following L1 overexpression in CRC cells is therefore a more general response of CRC cells to L1 transfection. Finally, the higher level of endogenous CTSD in cells after transfection with L1 could also be demonstrated by immunostaining of LS 174T CRC cells stably expressing L1 as compared to control pcDNA3-transfectd cells (Figure 1G).

### Modulation of CTSD levels in L1-expressing CRC cells affects their growth, motile and tumorigenic properties

**Figure 2:**
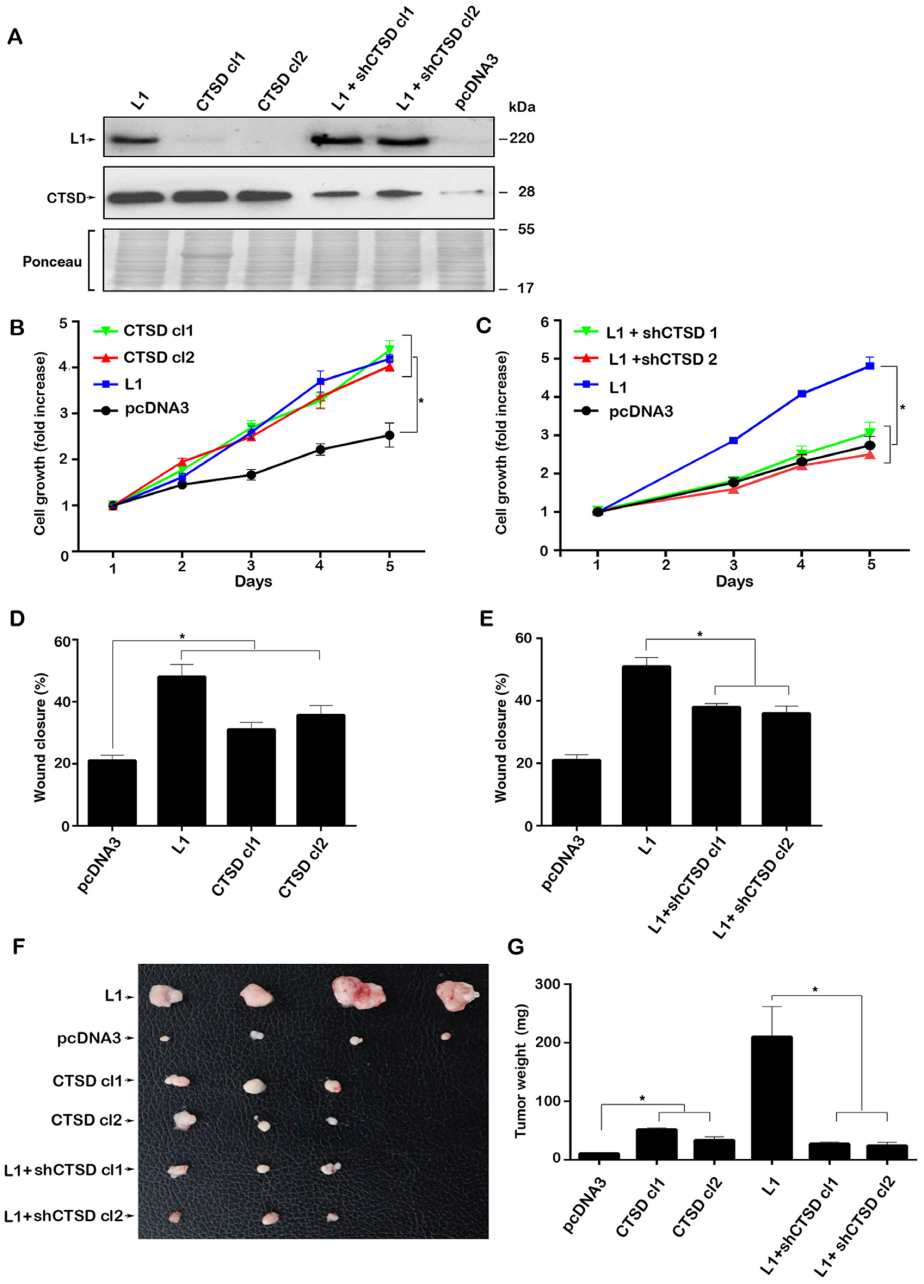
Modulating the expression of CTSD in CRC cells affects cell growth, motility and tumorigenesis. (**A**) Clones of LS 174T cells stably transfected with CTSD (CTSD cl1 and cl2), control pcDNA3 and L1-transfected LS 174T cells in which endogenous levels of CTSD were suppressed by shRNA (L1+shCTSD cl1 and cl2) were isolated. The expression of the relevant proteins was determined by western immunoblotting. (**B**) The proliferation in 0.1% serum of CTSD-overexpressing cells was compared to that of cells transfected with L1, or with pcDNA3. (**C**) The proliferation of LS 174T expressing L1+shCTSD cell clones was compared to that of cells expressing L1, or pcDNA3, over 5 days. (**D, E**) The motility of the LS 174T cell clones described in (A) was determined by the “scratch wound” experiment 24 hours after introducing the artificial wound in triplicate samples for each cell clone. (**F, G**) The tumorigenic capacity of the cell clones described in (A) was determined 2 weeks after s.c injection into nude mice of 10^6^ cells for each clone. (F) The tumors were excised and photographed and their weight (G) was determined. ^*^ *P* < 0.05. Two-sided Student’s *t* test.

We wished to determine whether the changes conferred by L1 in CRC cell motility and tumorigenicity [3, 5] require CTSD. For this, we isolated LS 174T cell clones stably expressing L1 in which the endogenous levels of CTSD were suppressed by shRNA to CTSD (Figure 2A, L1+shCTSD cl1 and cl2, Supplementary Figure 1C and 1D). We also isolated CRC cell clones stably overexpressing CTSD (Figure 2A, CTSD cl1 and cl2, Supplementary Figure 1E and 1F) to analyze whether the effects conferred by L1 overexpression could be caused by increased CTSD expression in the absence of L1. The results summarized in Figure 2B demonstrate that the proliferation under stressful conditions (in the absence of serum) of LS 174T CRC cell clones stably overexpressing CTSD is significantly increased (Figure 2B). Furthermore, in L1-overexpressing CRC cell clones in which the endogenous levels of CTSD were suppressed, the proliferation of cells in the absence of serum was reduced to the level seen in control LS 174T cells (Figure 2C). These results suggest that the increase in cell proliferation conferred by L1 expression is partially due to the elevated CTSD levels in such cells.

When analyzing the effects of changes in CTSD levels in CRC cells lacking or expressing L1, we observed a similar effect on cell motility (by the “scratch wound” closure experiment) and tumor growth in mice upon s.c injection (Figure 2D–2G). Thus, CTSD overexpression resulted in a modest, yet significant, increase in LS 174T cell motility (Figure 2D) and the suppression of endogenous CTSD levels in CRC cells stably expressing L1, reduced their motility (Figure 2E). The injection of these CRC cell clones s.c into immunocompromised mice resulted in a small increase in tumor formation upon CTSD overexpression (Figure 2F, compare CTSD cl 1 and 2 to L1), while CTSD suppression in L1 expressing cells resulted in a marked reduction in tumorigenic capacity of these cells (Figure 2F and 2G, compare L1+shCTSD cl1 and cl2 to L1).

**Figure 3:**
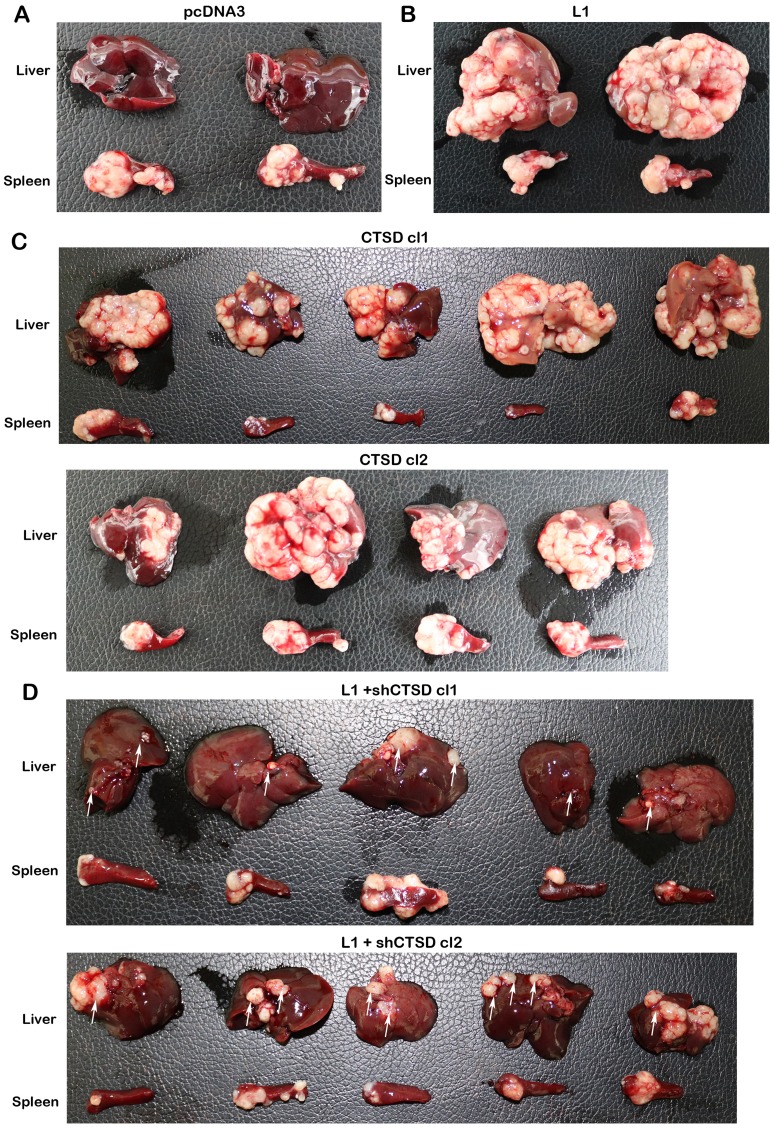
CTSD expression levels affect the metastatic ability of human CRC cells to the liver. The ability of the LS 174T cell clones described in (Figure 2A) to form liver metastases was determined by injecting 2 **×** 10^6^ cells into the spleen of nude mice for each cell line and excising the liver and spleen of such mice after 6 weeks. In control pcDNA3-transfected (**A**) and L1-transfected cells (**B**) the results with only two mice are shown. (**C**) CTSD overexpressing LS 174T cell clones (CTSD cl1 and cl2), and (**D**) L1+shCTSD cell clones (cl1 and cl2). The white areas in the liver tissue represent the metastatic lesions formed by the human CRC cells. The white arrows in (D) point to the much smaller metastatic foci formed when the levels of CTSD were suppressed in L1-expressing cells with shRNA to CTSD.

We have also studied the possible effects of CTSD on the ability of L1 to confer liver metastasis upon injecting the cells into the spleen [5] and following the formation of metastases in the liver. CRC cell clones stably overexpressing L1 very effectively formed liver metastases upon their injection into the spleen of mice (Figure 3B compare to 3A and [5]). The overexpression of CTSD alone also induced liver metastasis (Figure 3C), but to a lesser extent than L1 overexpression (compare Figure 3C to 3B, Supplementary Figure 2). CRC cells overexpressing L1 in which the endogenous CTSD levels were suppressed, had a dramatically reduced capacity to form metastases in the liver (Figure 3D), although they continued expressing L1 (Supplementary Figure 2). Taken together, the results described in Figures 2 and 3 demonstrated that while CTSD can promote the motile and tumorigenic capacity of CRC cells, CTSD is much less potent than L1 in conferring tumorigenic properties. On the other hand, in the context of L1-mediated effects on the tumorigenic and metastatic capacities of CRC cells, the increase in CTSD expression is essential for the L1-conferred tumorigenic properties.

### The increase in CTSD by L1 is mediated by enhanced Wnt/β-catenin signaling

**Figure 4:**
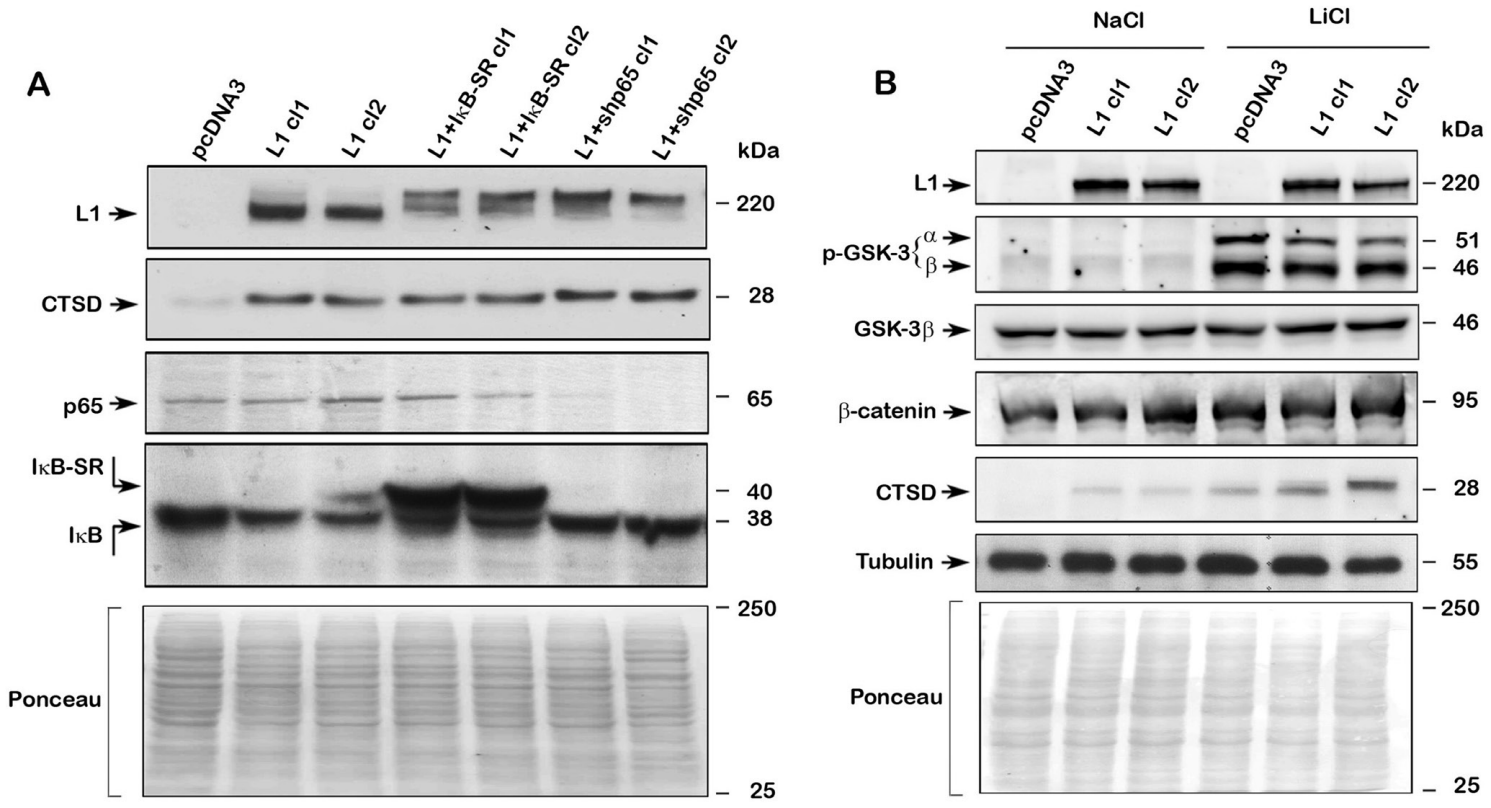
Regulation of CTSD expression by L1 does not involve NF-κB but is affected by Wnt/β-catenin signaling. (**A**) NF-κB signaling was blocked in L1-expressing CRC cell clones by stably expressing the mutant IκBα super repressor IκB-SR (L1+IκB-SR cl1 and cl2), or by suppressing the NF-κB subunit p65 using shp65RNA (L1+shp65 cl1 and cl2). The levels of endogenous CTSD and NF-κB signaling proteins were determined in these cell clones by western blotting with the appropriate antibodies. (**B**) Treatment with 30 mM LiCl was used to enhance β-catenin-TCF transactivation (treatment with NaCl served as control) and the level of CTSD was determined in pcDNA3 and L1-expressing LS 174T cell clones. The levels of phosphorylated GSK-3, total β-catenin and total GSK-3 were determined. Tubulin and Ponceau staining served as controls for equal loading of the gels. Note that treatment with L1Cl resulted in the elevation of the inactive p-GSK3α and β and increased CTSD levels, both in pcDNA3 and L1-expressing cells, suggesting that β-catenin-TCF signaling is involved in CTSD expression. No changes in total β-catenin and GSK-3 levels were detected. Inhibition of NF-κB signaling had no effect on CTSD levels in L1-expressing cells.

In previous studies we have shown that L1 exerts its downstream effects by signaling through the NF-κB pathway [12, 19]. We have therefore analyzed the levels of CTSD in L1-overexpressing CRC cell clones in which the signaling by NF-κB was blocked, either by expressing the IκBα super repressor (IκB-SR), or by reducing the level of the p65 NF-κB subunit using shRNA to p65 (Figure 4A). The inhibition of NF-κB signaling by these methods had no effect on the induction of CTSD in L1-overexpressing CRC cells (Figure 4A), suggesting that L1 induces CTSD via different signaling pathways.

We have recently demonstrated that L1 can induce downstream targets also by enhancing β-catenin localization to the nucleus and increasing β-catenin-TCF transactivation [20]. Since inhibiting GSK3 increases β-catenin-TCF transactivation [21], we have used LiCl to inhibit GSK3 and analyzed CTSD levels in L1-expressing LS 174T cells. The results summarized in Figure 4B demonstrate that LiCl (an inhibitor of GSK3 activity), but not NaCl, increased the inhibiting phosphorylation of GSK3α and 3β (Figure 4B, p-GSK-3α and β), and in LiCl-treated cells there was a further increase in CTSD levels in L1-expressing CRC cells (Figure 4B), implying that Wnt/β-catenin signaling could be the mechanism responsible for increased CTSD expression in LS 174T cells. We did not observe in this experiment a significant change in the total levels of either GSK3β or β-catenin (Figure 4B).

### CTSD is expressed in CRC cells and in the invasive stroma of human CRC tissue

**Figure 5:**
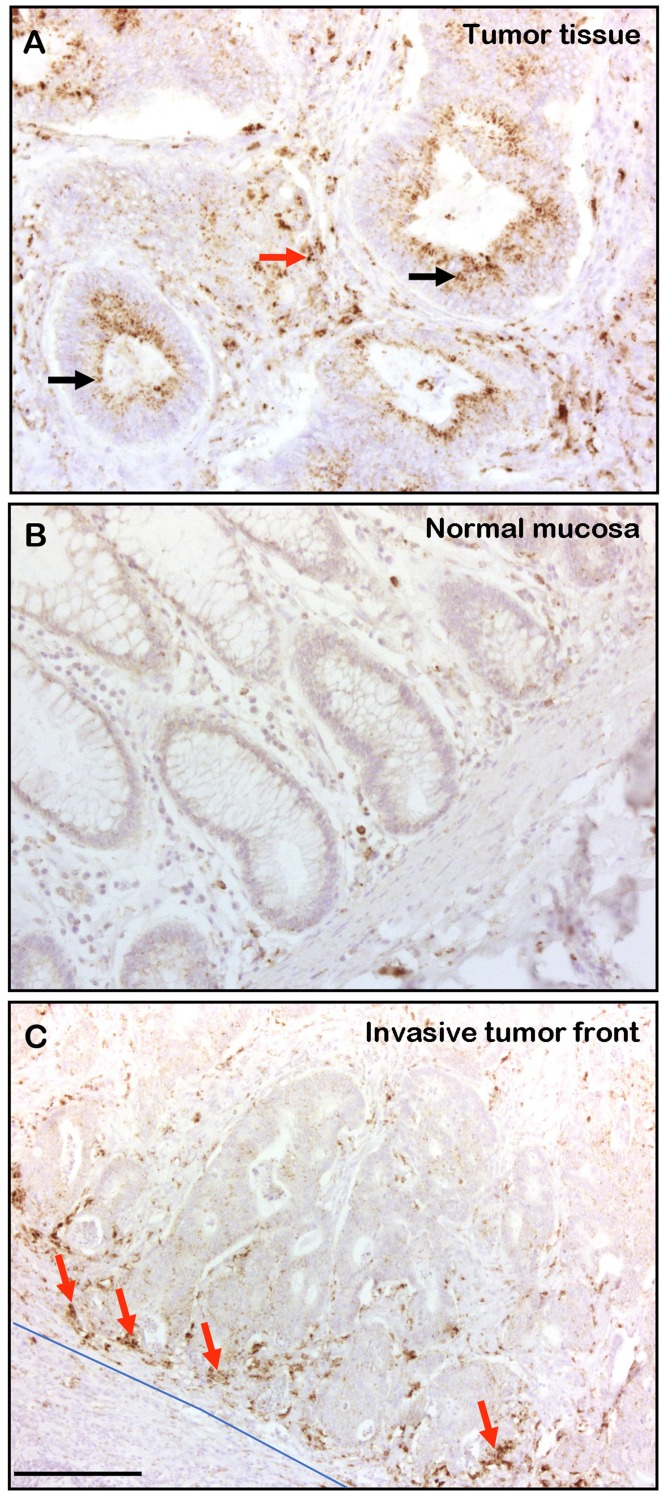
CTSD is expressed in human CRC tissue and in invasive areas of the stroma. Formaldehyde-fixed and paraffin embedded human CRC samples were analyzed by immunohistochemistry for CTSD presence. (**Α**) In 30 out of 38 samples the CRC tissue samples displayed strong CTSD staining of the epithelial tissue (black arrows) and, in addition, all CRC tissue samples were positive for CTSD in the stroma (red arrow). (**B**) The normal colon mucosa was not stained with anti CTSD antibody. (**C**) The invasive stromal area (blue line) was often strongly stained for CTSD (red arrows). The bar in (C) represents 300 μm.

We wished to determine the distribution of CTSD in human CRC tissue. Analysis of 38 human CRC cases revealed that 78% of the tumors displayed strong staining of the epithelial tumor tissue (Figure 5A, black arrows), while no significant staining was observed in the normal mucosa (Figure 5B). In addition, in all cases analyzed, the stromal tissue of CRC was strongly stained for CTSD (Figure 5A, red arrow), especially in the more invasive areas of the tumor tissue (Figure 5C, blue line and red arrows). These results indicate that CTSD expression is increased in CRC tissue with preference of the more invasive areas of the tumor thereby contributing to tumor progression.

## DISCUSSION

In previous studies, using gene arrays, we identified genes and signaling pathways that are involved in conferring enhanced tumorigenesis and metastasis in CRC cells by the Wnt/β-catenin target gene L1 [10]. In this study, we have used a proteomic approach to identify proteins in the secretome of L1-overexpressing CRC cells whose levels are increased after L1 expression and that might be involved in the L1-mediated promotion of tumor development. We identified the lysosomal and secreted aspartate protease CTSD and focused on studying its role in CRC development, since it was reported that CTSD is expressed in a wide variety of tumor types including breast cancer [22–28], prostate cancer [29], gastric cancer [30], osteosarcoma pulmonary metastases, bone cancer [31], ovarian cancer [32] and in liver metastases of colon adenocarcinoma [33–35]. We found that in addition to the increased abundance of CTSD in the secretome of L1-overexpressing CRC cells, CTSD RNA and protein levels were also higher in CRC cell lines overexpressing L1. Moreover, the stable expression of CTSD in CRC cells was sufficient to promote cell proliferation under stress (in serum-free medium) and the motility, tumorigenesis and liver metastasis by these cells. These effects of CTSD were similar to those conferred by L1 overexpression, although the L1-mediated effects on tumorigenesis were much stronger, suggesting that additional genes/proteins (besides CTSD) are involved in the effects conferred by L1 in CRC cells. The link between L1-signaling and CTSD was demonstrated by the experiments in which endogenous CTSD levels were suppressed in L1-overexpressing CRC cells. This resulted in a much-reduced cell proliferation, tumorigenesis and liver metastasis. These results suggest that increased CTSD expression by L1 overexpression is a necessary step in CRC tumor progression.

Previously, we have identified several signaling pathways involved in the downstream induction of genes by L1 overexpression in CRC cells, including NF-κB [12, 19], STAT-1 [14] and Wnt/β-catenin signaling [20]. In this study, we found that the induction of CTSD in L1-expressing CRC cells involves Wnt/β-catenin signaling, since by stimulating this pathway with the GSK3 inhibitor LiCl, we observed an increase in CTSD expression, while suppressing NF-κB signaling had no effect on CTSD levels.

We detected CTSD in the majority (~80%) of human CRC epithelial cells and in the stromal tissue of all CRC samples examined. In particular, CTSD was observed in the invasive front of the CRC tissue, implying that it plays a key role in CRC progression. These results on CTSD localization in CRC cells are in agreement with observations in breast epithelial cancer where CTSD was detected in both stromal and epithelial tissue [26].

The mechanism/s by which CTSD promotes tumorigenesis are still unclear and suggest both mitogenic, paracrine effects on stromal cells in the tumor environment and an interaction with a yet to be detected cell surface membrane receptor in the carcinoma cells. In addition, CTSD can act by digesting the extracellular matrix, releasing growth factors and promoting both tumor growth and invasion [15].

Taken together, the present study further highlights the importance of CTSD in human CRC progression and marks this protease as a therapeutic target for curing invasive CRC.

## MATERIALS AND METHODS

### Cell culture, transfections, cell proliferation and motility assays

The cell lines LS 174T and HCT 116 were grown as previously described [3]. LS 174T-L1, LS 174T-CTSD, LS 174T-IκB-SR, HCT 116-L1 cells were maintained in medium containing neomycin (1 mg/ml), and LS 174T-L1+shCTSD and LS 174T-L1+shp65 cells were cultured in medium containing both neomycin (1 mg/ml) and puromycin (10 μg/ml). Transfection of LS 174T cells was performed using Lipofectamine^TM^ 2000 (ThermoFisher Scientific, MA, USA) according to the manufacturer’s instructions. Five thousand cells were seeded in 96-well plates in medium containing 0.1% serum and cell growth was determined by the XTT (sodium 2,3-bis(2-methoxy-4-nitro-5-sulfophenyl)-5-[(phenylamino)-carbonyl]-2H-tetrazolium) cell viability assays (Biological Industries, Israel). Cell motility was assessed by the artificial “scratch-wound” closure assay as described [12]. To inhibit glycogen synthase kinase 3 beta (GSK3β) the cells were incubated for 24 hours with 30 mM LiCl. Treatment with 30 mM NaCl served as control.

### Plasmids

The CTSD expression vectors were obtained from Dr. E. Liaudet-Coopman (IRCM, Montpellier, France). CTSD shRNA was prepared in pSUPER.puro by following the manufacturer’s instructions (pSUPER.puro RNAi System, OligoEngine, WA, USA) using the target sequences as described in Supplementary Table 1.

### Immunoblotting and immunofluorescence

Immunoblotting was carried out using the following antibodies: Rabbit anti-L1 (a gift from Dr. V. Lemmon, University of Miami, FL, USA) at 1:8,000 dilution, mouse anti-CTSD MAB 610800 (BD Biosciences, USA) was diluted 1:1,000, rabbit anti-phospho-IκBα #2859 (Cell Signaling Technologies Inc., MA, USA) was diluted 1:1,000, rabbit anti β-catenin C2206 (Sigma-Aldrich Israel ltd., Israel) was diluted 1:200, phospho-GSK-3α/β (Ser21/9) from Cell Signaling Technologies, was diluted 1:1,000, GSK-3β, #9832 antibody from Cell Signaling Technologies was diluted 1:2,000 and mouse anti-β-tubulin (Sigma-Aldrich Israel ltd, Israel) was diluted 1:100,000. Cells were lysed in RIPA buffer containing 150 mM NaCl, 50 mM Tris pH 7.5, 1 mM EDTA, 1% sodium deoxycholate, 1% NP-40, and 0.1% sodium dodecyl sulfate (SDS), supplemented with the complete protease inhibitor cocktail (Roche, Switzerland). Cell lysates were loaded in Laemmli’s buffer (20% glycerol, 3% DTT, 6% SDS, 0.1% bromophenol blue, 62.5 mM Tris pH 6.8).

Western blots were developed using the ECL method (Amersham Biosciences, UK). To analyze proteins secreted into the cell culture medium, cells were grown until confluency and the medium was changed to medium containing 0.1% FCS for 24 hours. The cells were lysed with RIPA buffer. The medium was collected and centrifuged at 4500 rpm for 10 minutes to sediment cell debris. The medium supernatant was mixed with cold 100% ethanol at a ratio of 1:9 and proteins were sedimented after 24 hours at –80°C by centrifugation at 15,000 RPM for 30 minutes. BCA was used to determine protein levels and to enable equal loading for western blotting.

For immunofluorescence, cells cultured on glass coverslips were permeabilized with 0.5% Triton X-100 in 4% PFA for 2 minutes. The same primary antibodies against CTSD were used for immunofluorescence as for immunoblotting. The secondary antibody was Cy3-labeled goat anti-mouse IgG (Jackson ImmunoResearch Laboratories, PA, USA) diluted 1:10,000 in PBS. Nuclei were stained using 5 µg/ml 4′-6-diamidino-2-phenylindole (DAPI, Sigma-Aldrich, MO, USA). Images were acquired by using the Eclipse E1000, Nikon objective 60X/1.4 NA with a camera (ORCA-ER; Hamamatsu) and the volocity acquisition software (PerkinElmer, MAS, USA).

### Immunohistochemistry

Immunohistochemistry was carried out on paraffin-embedded human colorectal adenocarcinomas as previously described [3]. CTSD was stained using the monoclonal mouse anti human CTSD antibody (610800 BD Biosciences, USA), diluted 1:50. The streptomycin/AB system was employed to detect antibody binding according to the manufacturer’s protocol (Dako, Germany). Sections were counterstained with Hemalaun (Merck, Germany).

### Quantitative RT-PCR

Total RNA was isolated from cells using the EZ-RNA II Total RNA isolation kit (Biological Industries, Israel) according to the manufacturer’s protocol. First strand cDNA was synthesized by using the Superscript^TM^ First Strand Synthesis System (ThermoFisher Scientific, MA, USA) using the primers described in Supplementary Table 1. Real-Time PCR was performed as described [12]. Data analysis was performed using the ΔΔC_T_ method with the StepOne software v2.3 (ThermoFisher Scientific, MA, USA).

### Mass spectrometry

LS 174T cells stably transfected with either L1 or pcDNA3 were grown until confluency. Cells were further grown in phenol red-free RPMI 1640 and 0.1% FCS for 24 hours. The medium from the cell cultures was collected and centrifuged at 4,500 RPM for 10 minutes to sediment cellular debris from the medium. The medium was subsequently analyzed by liquid chromatography and tandem mass spectrometry at the Mass Spectrometry Facility of the Nancy and Stephen Grand Israel National Center for Personalized Medicine at the Weizmann Institute of Science [36]. Proteins with high presence in the medium were detected by matching peptide sequences to the uniport database using the Mascot program [37].

### Tumor growth and metastasis assays

Tumor growth subcutaneously was induced as described [12]. The ability of cells to metastasize was determined by injecting 2.5 × 10^6^ cells in 20 µl PBS into the distal tip of the spleen of 6 weeks-old male nude mice. Mice were anesthetized by peritoneal injection of 1 μl/mg xylazine (20 mg/ml) and 1 μl/mg ketamine (100 mg/ml). Animals were sacrificed after 7 weeks, and primary tumor formation in the spleen and metastasis formation in the liver were examined. Tumor area was calculated using the ImageJ software [38].

### Statistical analysis

In the mouse metastasis experiments, statistical significance between pcDNA3, CTSD cl1 and CTSD cl2 were determined by the Wilcoxon Signed-rank Test, while the significance between L1, L1+shCTSD1 and L1+shCTSD2 were determined by the Fisher’s exact test. The significance of qRT-PCR comparisons for RNA levels and wound closure experiments was determined by ANOVA. The significance of all other comparisons was determined by ANOVA. A *P* value of < 0.05 was considered significant.

## SUPPLEMENTARY MATERIALS



## Abbreviations

CTSD: cathepsin D
GSK3: glycogen synthase kinase 3
CRC: colorectal cancer
TCF: T-cell factor
L1: L1 cell adhesion molecule
NF-κB: nuclear factor kappa B
IκBα: inhibitor of kappa B alpha

## References

[1] Bienz M , Clevers H . Linking colorectal cancer to Wnt signaling. Cell. 2000; 103:311–320. 10.1016/S0092-8674(00)00122-7. 11057903

[2] Conacci-Sorrell M , Zhurinsky J , Ben-Ze’ev A . The cadherin-catenin adhesion system in signaling and cancer. J Clin Invest. 2002; 109:987–991. 10.1172/JCI15429. 11956233 PMC150951

[3] Gavert N , Conacci-Sorrell M , Gast D , Schneider A , Altevogt P , Brabletz T , Ben-Ze’ev A . L1, a novel target of beta-catenin signaling, transforms cells and is expressed at the invasive front of colon cancers. J Cell Biol. 2005; 168:633–642. 10.1083/jcb.200408051. 15716380 PMC2171754

[4] Conacci-Sorrell ME , Ben-Yedidia T , Shtutman M , Feinstein E , Einat P , Ben-Ze’ev A . Nr-CAM is a target gene of the beta-catenin/LEF-1 pathway in melanoma and colon cancer and its expression enhances motility and confers tumorigenesis. Genes Dev. 2002; 16:2058–2072. 10.1101/gad.227502. 12183361 PMC186445

[5] Gavert N , Sheffer M , Raveh S , Spaderna S , Shtutman M , Brabletz T , Barany F , Paty P , Notterman D , Domany E , Ben-Ze’ev A . Expression of L1-CAM and ADAM10 in human colon cancer cells induces metastasis. Cancer Res. 2007; 67:7703–7712. 10.1158/0008-5472.CAN-07-0991. 17699774

[6] Lemmon V , Farr KL , Lagenaur C . L1-mediated axon outgrowth occurs via a homophilic binding mechanism. Neuron. 1989; 2:1597–1603. 10.1016/0896-6273(89)90048-2. 2627381

[7] Wong EV , Kenwrick S , Willems P , Lemmon V . Mutations in the cell adhesion molecule L1 cause mental retardation. Trends Neurosci. 1995; 18:168–172. 10.1016/0166-2236(95)93896-6. 7778187

[8] Cohen NR , Taylor JS , Scott LB , Guillery RW , Soriano P , Furley AJ . Errors in corticospinal axon guidance in mice lacking the neural cell adhesion molecule L1. Curr Biol. 1998; 8:26–33. 10.1016/S0960-9822(98)70017-X. 9427628

[9] Haase G , Gavert N , Brabletz T , Ben-Ze’ev A . A point mutation in the extracellular domain of L1 blocks its capacity to confer metastasis in colon cancer cells via CD10. Oncogene. 2017; 36:1597–1606. 10.1038/onc.2016.329. 27641335

[10] Haase G , Gavert N , Brabletz T , Ben-Ze’ev A . The Wnt target gene L1 in colon cancer invasion and metastasis. Cancers (Basel). 2016; 8:E48. 10.3390/cancers8050048. 27187476 PMC4880865

[11] Gavert N , Shvab A , Sheffer M , Ben-Shmuel A , Haase G , Bakos E , Domany E , Ben-Ze’ev A . c-Kit is suppressed in human colon cancer tissue and contributes to L1-mediated metastasis. Cancer Res. 2013; 73:5754–5763. 10.1158/0008-5472.CAN-13-0576. 24008320

[12] Ben-Shmuel A , Shvab A , Gavert N , Brabletz T , Ben-Ze’ev A . Global analysis of L1-transcriptomes identified IGFBP-2 as a target of ezrin and NF-kappaB signaling that promotes colon cancer progression. Oncogene. 2013; 32:3220–3230. 10.1038/onc.2012.340. 22869145

[13] Shvab A , Haase G , Ben-Shmuel A , Gavert N , Brabletz T , Dedhar S , Ben-Ze’ev A . Induction of the intestinal stem cell signature gene SMOC-2 is required for L1-mediated colon cancer progression. Oncogene. 2016; 35:549–557. 10.1038/onc.2015.127. 25915847

[14] Shapiro B , Tocci P , Haase G , Gavert N , Ben-Ze’ev A . Clusterin, a gene enriched in intestinal stem cells, is required for L1-mediated colon cancer metastasis. Oncotarget. 2015; 6:34389–34401. 10.18632/oncotarget.5360. 26399194 PMC4741460

[15] Liaudet-Coopman E , Beaujouin M , Derocq D , Garcia M , Glondu-Lassis M , Laurent-Matha V , Prebois C , Rochefort H , Vignon F . Cathepsin D: newly discovered functions of a long-standing aspartic protease in cancer and apoptosis. Cancer Lett. 2006; 237:167–179. 10.1016/j.canlet.2005.06.007. 16046058

[16] Benes P , Vetvicka V , Fusek M . Cathepsin D--many functions of one aspartic protease. Crit Rev Oncol Hematol. 2008; 68:12–28. 10.1016/j.critrevonc.2008.02.008. 18396408 PMC2635020

[17] Chen S , Dong H , Yang S , Guo H . Cathepsins in digestive cancers. Oncotarget. 2017; 8:41690–41700. 10.18632/oncotarget.16677. 28402938 PMC5522190

[18] Turk V , Kos J , Turk B . Cysteine cathepsins (proteases)--on the main stage of cancer? Cancer Cell. 2004; 5:409–410. 10.1016/S1535-6108(04)00117-5. 15144947

[19] Gavert N , Ben-Shmuel A , Lemmon V , Brabletz T , Ben-Ze’ev A . Nuclear factor-kappaB signaling and ezrin are essential for L1-mediated metastasis of colon cancer cells. J Cell Sci. 2010; 123:2135–2143. 10.1242/jcs.069542. 20501702 PMC4481617

[20] Basu S , Gavert N , Brabletz T , Ben-Ze’ev A . The intestinal stem cell regulating gene ASCL2 is required for L1-mediated colon cancer progression. Cancer Lett. 2018; 424:9–18. 10.1016/j.canlet.2018.03.022. 29551399

[21] Stamos JL , Weis WI . The beta-catenin destruction complex. Cold Spring Harb Perspect Biol. 2013; 5:a007898. 10.1101/cshperspect.a007898. 23169527 PMC3579403

[22] Zhang M , Wu JS , Yang X , Pang X , Li L , Wang SS , Wu JB , Tang YJ , Liang XH , Zheng M , Tang YL . Overexpression cathepsin D contributes to perineural invasion of salivary adenoid cystic carcinoma. Front Oncol. 2018; 8:492–492. 10.3389/fonc.2018.00492. 30430081 PMC6220369

[23] Rochefort H , Cavailles V , Augereau P , Capony F , Maudelonde T , Touitou I , Garcia M . Overexpression and hormonal regulation of pro-cathepsin D in mammary and endometrial cancer. J Steroid Biochem. 1989; 34:177–182. 10.1016/0022-4731(89)90080-0. 2626016

[24] Capony F , Rougeot C , Cavailles V , Rochefort H . Estradiol increases the secretion by MCF7 cells of several lysosomal pro-enzymes. Biochem Biophys Res Commun. 1990; 171:972–978. 10.1016/0006-291x(90)90779-m. 2222457

[25] Roger P , Montcourrier P , Maudelonde T , Brouillet JP , Pages A , Laffargue F , Rochefort H . Cathepsin D immunostaining in paraffin-embedded breast cancer cells and macrophages: correlation with cytosolic assay. Hum Pathol. 1994; 25:863–871. 10.1016/0046-8177(94)90004-3. 8088760

[26] Tetu B , Brisson J , Lapointe H , Wang CS , Bernard P , Blanchette C . Cathepsin D expression by cancer and stromal cells in breast cancer: an immunohistochemical study of 1348 cases. Breast Cancer Res Treat. 1999; 55:137–147. 10.1023/A:1006140213493. 10481941

[27] Berchem G , Glondu M , Gleizes M , Brouillet JP , Vignon F , Garcia M , Liaudet-Coopman E . Cathepsin-D affects multiple tumor progression steps *in vivo*: proliferation, angiogenesis and apoptosis. Oncogene. 2002; 21:5951–5955. 10.1038/sj.onc.1205745. 12185597

[28] Ashraf Y , Mansouri H , Laurent-Matha V , Alcaraz LB , Roger P , Guiu S , Derocq D , Robin G , Michaud HA , Delpech H , Jarlier M , Pugniere M , Robert B , et al. Immunotherapy of triple-negative breast cancer with cathepsin D-targeting antibodies. J Immunother Cancer. 2019; 7:29. 10.1186/s40425-019-0498-z. 30717773 PMC6360707

[29] Vetvicka V , Vetvickova J , Fusek M . Role of procathepsin D activation peptide in prostate cancer growth. Prostate. 2000; 44:1–7. 10.1002/1097-0045(20000615)44:13::AID-PROS13E3.0.CO2-4. 10861751

[30] Yang L , Cui M , Zhang L , Song L . FOXM1 facilitates gastric cancer cell migration and invasion by inducing Cathepsin D. Oncotarget. 2017; 8:68180–68190. 10.18632/oncotarget.19254. 28978107 PMC5620247

[31] Gemoll T , Epping F , Heinrich L , Fritzsche B , Roblick UJ , Szymczak S , Hartwig S , Depping R , Bruch HP , Thorns C , Lehr S , Paech A , Habermann JK . Increased cathepsin D protein expression is a biomarker for osteosarcomas, pulmonary metastases and other bone malignancies. Oncotarget. 2015; 6:16517–16526. 10.18632/oncotarget.4140. 26203049 PMC4599286

[32] Pranjol MZ , Gutowski N , Hannemann M , Whatmore J . The potential role of the proteases cathepsin D and cathepsin L in the progression and metastasis of epithelial ovarian cancer. Biomolecules. 2015; 5:3260–3279. 10.3390/biom5043260. 26610586 PMC4693277

[33] Oh-e H , Tanaka S , Kitadai Y , Shimamoto F , Yoshihara M , Haruma K . Cathepsin D expression as a possible predictor of lymph node metastasis in submucosal colorectal cancer. Eur J Cancer. 2001; 37:180–188. 10.1016/S0959-8049(00)00348-8. 11166144

[34] Kirana C , Shi H , Laing E , Hood K , Miller R , Bethwaite P , Keating J , Jordan TW , Hayes M , Stubbs R . Cathepsin D expression in colorectal cancer: from proteomic discovery through validation using western blotting, immunohistochemistry, and tissue microarrays. Int J Proteomics. 2012; 2012:245819. 10.1155/2012/245819. 22919486 PMC3420108

[35] Mehrotra S , Wickremesekera SK , Brasch HD , Van Schaijik B , Marsh RW , Tan ST , Itinteang T . Expression and localization of cathepsins B, D and G in cancer stem cells in liver metastasis from colon adenocarcinoma. Front Surg. 2018; 5. 10.3389/fsurg.2018.00040. 30177970 PMC6110174

[36] Loewenstein S , Lubezky N , Nizri E , Zemel M , Levin Y , Savidor A , Sher O , Klausner JM , Lahat G . Adipose-induced retroperitoneal soft tissue sarcoma tumorigenesis: A potential crosstalk between sarcoma and fat cells. Mol Cancer Res. 2016; 14:1254–1265. 10.1158/1541-7786.MCR-16-0131. 27621268

[37] Perkins DN , Pappin DJ , Creasy DM , Cottrell JS . Probability-based protein identification by searching sequence databases using mass spectrometry data. Electrophoresis. 1999; 20:3551–3567. 10.1002/(SICI)1522-2683(19991201)20:183C3551::AID-ELPS35513E3.0.CO2-2. 10612281

[38] Chang J , Erler JT . Quantification of lung metastases from *in vivo* mouse models. Adv Exp Med Biol. 2016; 899:245–251. 10.1007/978-3-319-26666-4_14. 27325271

