# Supplementary material for: Increased expression of cathepsin D is required for L1-mediated colon cancer progression
Source: Oncotarget. 2019 Aug 27;10(50):5217–28. doi: 10.18632/oncotarget.27155 (PMC6718269; doi:10.18632/oncotarget.27155)
Supplement: Supplementary file 1 [file oncotarget-10-5217-s001.pdf]

Increased expression of cathepsin D is required for L1-mediated colon cancer progression

SUPPLEMENTARY MATERIALS

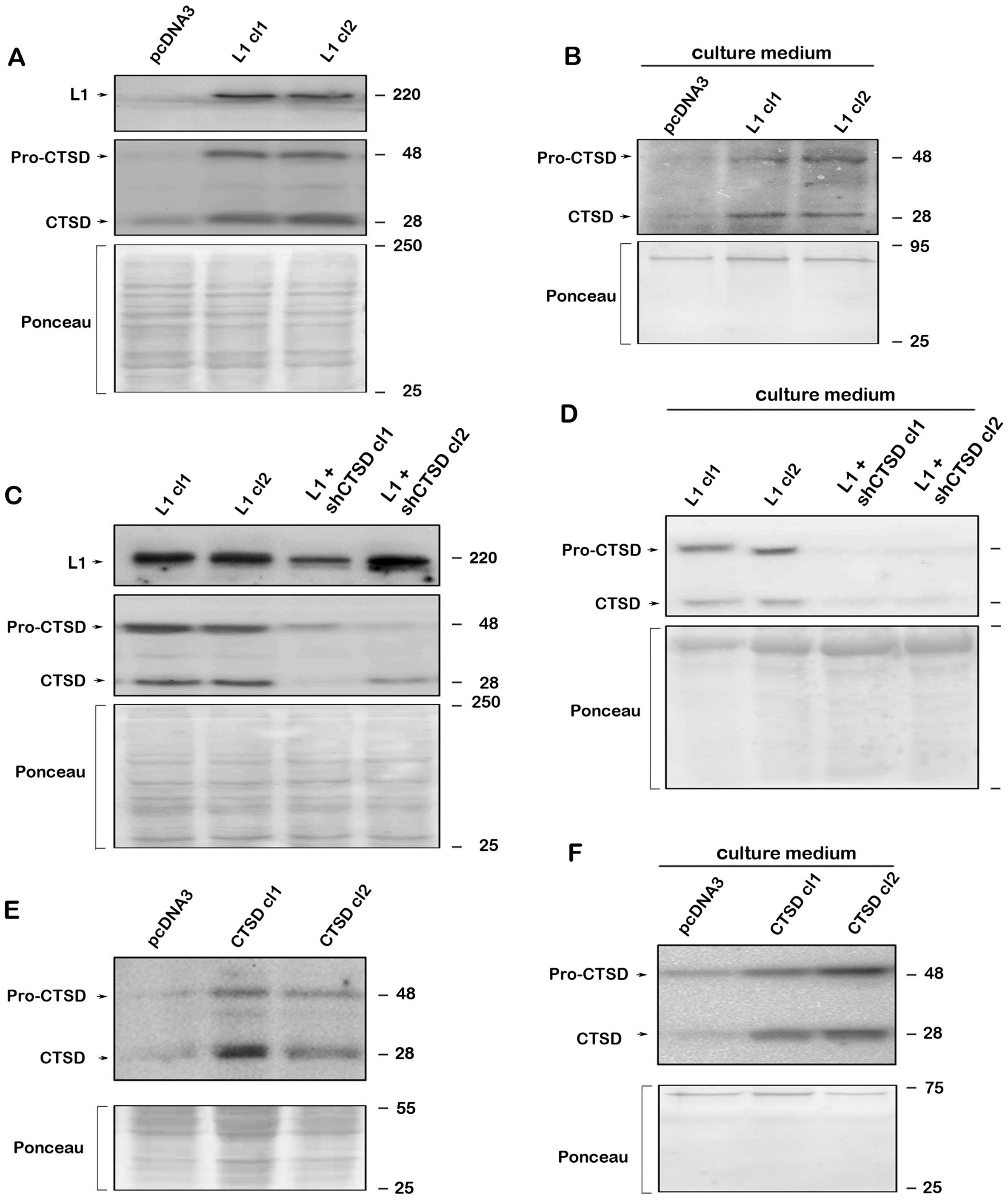

Supplementary Figure 1: Coordinated regulation of Pro-CTSD and CTSD in LS 174T CRC cell clones. CTSD and Pro-CTSD levels are increased in the cell layer (**A**) and in the culture medium (**B**) of L1-overexpressing CRC cell clones. The suppression of CTSD in L1-overexpressing CRC cell clones with shCTSD is followed by decreased CTSD and Pro-CTSD in the cell layer (**C**) and in the culture medium (**D**). CTSD overexpression in CRC cells resulted in increased Pro-CTSD and CTSD in the cell layer (**E**) and in the culture medium (**F**).

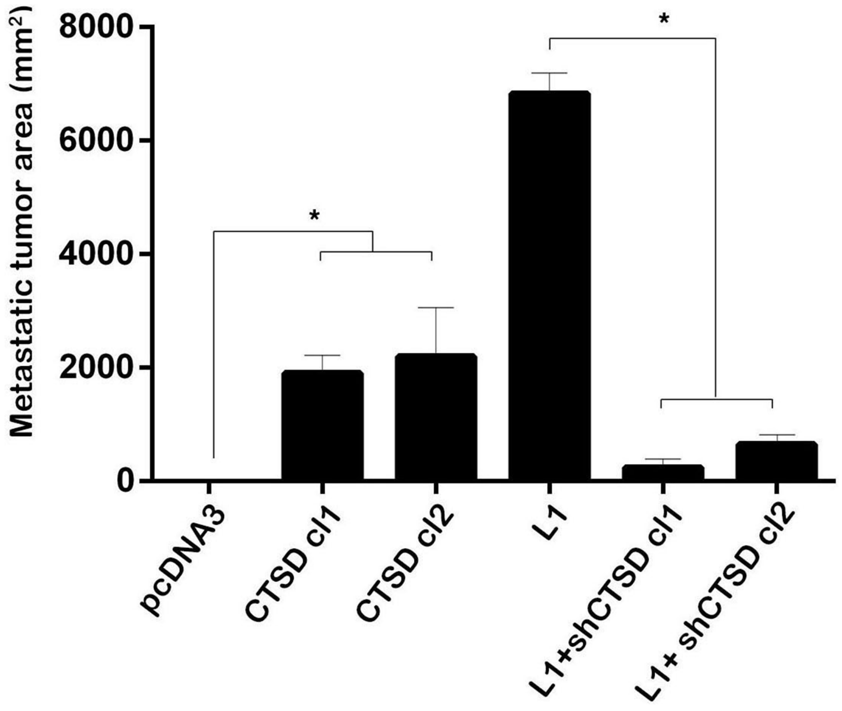

Supplementary Figure 2: The expression of CTSD is required for increased liver metastasis conferred by L1 in CRC cell clones. LS 174T CRC cells clones stably expressing pcDNA3, L1, CTSD (cl1 and cl2), and L1+shCTSD (cl1 and cl2) were injected into the spleen of mice and the development of metastases in the liver was determined by measuring the area of metastatic foci formed in the liver as described in Materials and Methods.

Supplementary Table 1: Sequences of shRNA targeted against CTSD RNA

Name	Primer Sequence
shCTSD1	GATCCCCAAGTGGTGGACCAGAACATCTTCAAGAGAGATGTTCTGGTCCACCAGCTTTTTTTA
shCTSD2	GATCCCCAATGGTACCTCGTTTGACTTCAAAGAGAAGTCAACGAGGTACCATTCTTTTTA
shCTSD3	GATCCCCGTATTACAAGGGTTCTCTGTCAAGAGACAGAGAACCCTTTGTAATACTTTTTTA
